# A Structural Shift in the Readability of Ophthalmology Abstracts in Late 2023

**DOI:** 10.1016/j.xops.2026.101337

**Published:** 2026-07-28

**Authors:** Jonas Neubauer, Karl Ulrich Bartz-Schmidt, Faik Gelisken

**Affiliations:** Department of Ophthalmology, Eberhard-Karls University, Tübingen, Germany

**Keywords:** Abstract readability, Scientific writing, Ophthalmic research, AI-assisted writing, Bibliometric analysis

## Abstract

**Objective:**

To assess long-term trends in the readability of ophthalmology abstracts and to determine whether a structural change occurred during the study period.

**Design:**

Bibliometric longitudinal analysis.

**Subjects:**

A total of 198, 201 unique English-language abstracts published between January 2000 and September 2025 in 67 PubMed-indexed ophthalmology journals.

**Methods:**

Abstract readability was quantified using the Flesch Reading Ease score. Temporal trends were analyzed using segmented regression with data-driven breakpoint selection. Secondary analyses included additional validated readability indices and linguistic features (including lexical density, lexical diversity, and clause density). Differences before and after the breakpoint were assessed using nonparametric tests.

**Main Outcome Measures:**

Changes in abstract readability over time, primarily measured by the Flesch Reading Ease score.

**Results:**

Readability improved gradually from 2000 until 2023, followed by a marked decline thereafter. Segmented regression identified a structural breakpoint in October 2023, after which readability declined sharply, with mean Flesch Reading Ease scores decreasing by –0.222 (95% confidence interval, –0.257 to –0.204) points per month compared with a prebreakpoint increase of +0.008 (95% confidence interval, 0.007‑0.010) points per month (*P* < 0.001). Readability differed significantly between the pre-October and post-October 2023 periods (median, 30.74 [interquartile range, 30.39–31.06] vs. 28.35 [interquartile range, 27.29–29.54]; *P* < 0.001). Similar patterns were observed across multiple readability indices. The decline was accompanied by increases in syllables per word, proportion of polysyllabic words, lexical density, lexical diversity, and clause density, whereas words per sentence continued to decrease over time. Findings were consistent across journal impact strata and remained evident in within-author analyses.

**Conclusions:**

In this large longitudinal study of ophthalmology abstracts, readability improved for more than 2 decades before reversing sharply in late 2023. The decline was associated with increased lexical complexity and with indicators of greater syntactic complexity and was observed across journals and author subgroups. Although the causes of this shift cannot be determined from the present data, the findings indicate a meaningful change in the linguistic characteristics of ophthalmology abstracts and underscore the importance of maintaining readability in scientific communication

**Financial Disclosures:**

Proprietary or commercial disclosure may be found in the Footnotes and Disclosures at the end of this article.

Effective scientific communication is fundamental to the dissemination and interpretation of research findings and should enable readers to comprehend the main findings of a study quickly. Consequently, abstracts play an important role, as they are the first and sometimes only part of the publication read by researchers and clinicians.

Readability scores such as the Flesch Reading Ease describe the linguistic complexity of texts and reflect how easily readers can understand and engage with their content. Although they do not account for scientific rigor or conceptual depth, they influence how information is perceived, understood, and evaluated. Therefore, readability of scientific abstracts represents a key component of the accessibility of research findings.

Over recent decades, the style, structure, and complexity of scientific writing have changed in response to cultural and institutional influences.^1^ Currently, we are observing a significant technological transition, as in late 2022 large language models (LLMs) became accessible to the general public and are increasingly discussed in the context of scientific writing.^2^, ^3^, ^4^ Because publications typically do not disclose LLM usage, it has to be inferred indirectly. Recent approaches range from classifier-based models that differentiate between human-generated and LLM-generated texts to statistical analyses of lexical patterns, based on the observation that LLMs show distinct word usage compared to humans.^5^, ^6^, ^7^ Regardless of the methodology applied, studies consistently indicate that LLMs are now widely used in academic writing across multiple fields.^8^ Although LLM-based writing tools may improve efficiency and reduce language barriers, they may also alter stylistic and linguistic features of scientific text.^9^^,^^10^

To date, readability research in ophthalmology has primarily focused on patient education materials.^11^^,^^12^ In contrast, the readability of scientific abstracts—despite their central role in knowledge dissemination—has received little attention, and changes in readability within ophthalmic research remain largely unexplored.

In this study, we aimed to quantify temporal changes in the readability of ophthalmology abstracts over a 25-year period and to assess whether a structural change in readability occurred during the study period using validated readability metrics. In addition, we examined underlying linguistic features to better characterize potential shifts in writing style.

## Methods

The PubMed baseline data set and the daily updates released up to pubmed25n1603.xml were downloaded from the homepage of the National Center for Biotechnology Information. XML files were parsed to extract abstracts and associated metadata. English-language abstracts were included with a length of 250 to 4000 characters, and only publications from a curated list of 67 ophthalmology journals were retained (Supplementary, available at www.ophthalmologyscience.org), resulting in 234 331 abstracts. Duplicate entries were identified by PubMed Identifier (PMID). For records sharing the same PMID, abstracts were compared textually, and when both the abstract and PMID were identical, only the most recent version was retained. For the remaining duplicated PMIDs, the entry with the longer abstract was preserved. Finally, text processing followed the protocol described by González-Márquez et al and noninformative content such as copyright statements and publisher notices were hereby removed.^13^ The final data set comprised 198 201 unique abstracts published between January 1, 2000 and September 30, 2025.

Readability scores were calculated using the *textstat* Python library (version 0.7.2). For each abstract, the following metrics were computed: Flesch Reading Ease, Flesch Kincaid Grade Level, Gunning Fog Index, Dale Chall Readability Score, Coleman Liau Index, syllable count, polysyllabic word count, lexicon count, and sentence count.^14^ The Simple Measure of Gobbledygook Index was excluded from the analysis because it was originally validated on text samples of approximately 30 sentences and yields unstable estimates for short scientific abstracts.^15^

To further characterize linguistic changes, lexical density, lexical diversity, and syntactic complexity were quantified for each abstract using the spaCy natural language processing library (version 3.8.11, en_core_web_sm). Lexical density was defined as the proportion of nonstopword tokens among all tokens after removal of punctuation, whitespace, and numeric elements. Lexical diversity was assessed using the Measure of Textual Lexical Diversity, calculated bidirectionally and averaged per abstract. Syntactic complexity was approximated as the number of verb tokens per sentence, using verb counts as a proxy for clausal structure in English. Monthly mean values were analyzed using segmented linear regression with a prespecified breakpoint (October 2023), modeling level and slope changes.

To investigate the influence of style-associated vocabulary, the curated list of “style words” reported by Kobak et al was adopted (Supplementary Table S1, available at www.ophthalmologyscience.org).^5^ For each abstract, the frequency of these words per 1000 words was quantified.

Flesch Reading Ease scores were analyzed using segmented regression with an unknown breakpoint to detect changes in long-term trends. The breakpoint was estimated by comparing candidate models and selecting the model that minimized the Akaike Information Criterion, with a minimum of 12 months of data required on each side of the breakpoint. Models allowed for a change in slope while maintaining continuity at the breakpoint. Autocorrelation was addressed using heteroskedasticity-consistent and autocorrelation-consistent standard errors. Differences in readability between the 24 months before and after October 2023 and between journal impact strata (Q1–Q2 vs. Q3–Q4) were assessed using the Mann–Whitney U test.

For stratified analysis, the OpenAlex metadata were retrieved by linking records via PMID using the OpenAlex API (accessed on March 15, 2026). The country of the first author’s affiliation was obtained from OpenAlex metadata. Publications with multicountry affiliations for the first author were excluded. Countries were categorized as native English-speaking (United States, United Kingdom, Australia, Canada, New Zealand, and Ireland) or non-native English-speaking (all others). To assess within-author changes over time and account for repeated observations, a mixed-effects segmented regression model with a random intercept for author was fitted, restricted to authors who published both before and after October 2023. To assess whether changes in abstract format could confound temporal readability trends, abstracts were classified as structured or nonstructured using the XML-based abstract-section metadata.

Associations between style-word frequency and readability were assessed using Pearson correlation at the monthly level. *P* values <0.05 were considered significant. Ethics committee approval was not required for this study because all data analyzed were publicly available (Institutional Review Board of Eberhard-Karls University Tuebingen Project ID 011/2026A).

## Results

**Figure 1:**
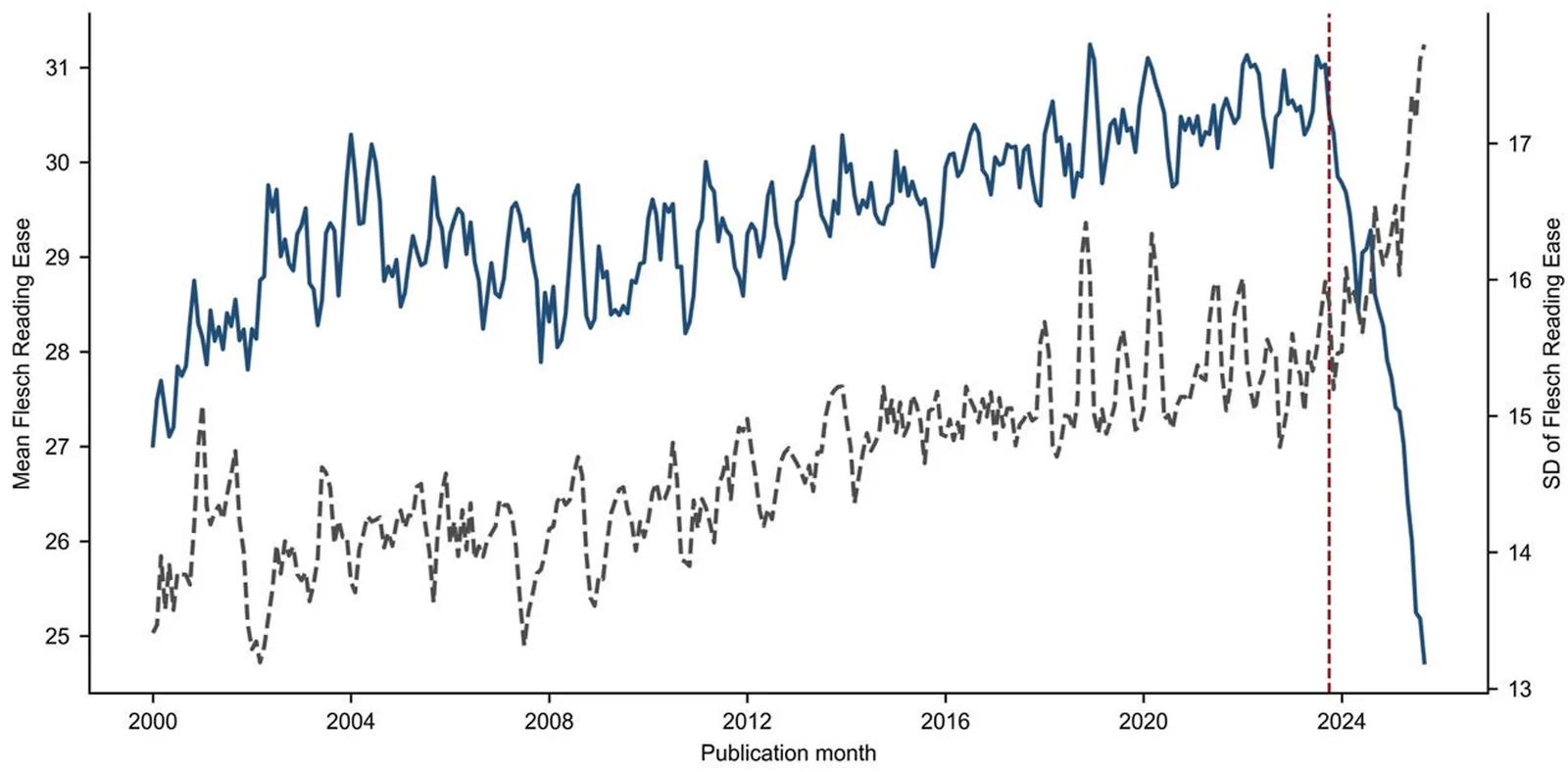
Trends and variability in readability of ophthalmology abstracts. Monthly mean Flesch Reading Ease (blue solid line, left y-axis) and corresponding standard deviations (gray dashed line, right y-axis) of ophthalmology abstracts. Lines represent centered 3-month rolling means. The vertical red dashed line indicates October 2023.

We analyzed 198 201 abstracts from 67 ophthalmology journals published between January 2000 and September 2025. Readability improved from 2000 to 2023, followed by a sharp decline thereafter (Fig 1). At the same time, the standard deviation, which had remained largely stable over many years, increased sharply as well (Fig 1).

Segmented regression identified a structural breakpoint in October 2023 (Fig 1). Prior to this breakpoint, mean Flesch Reading Ease scores increased slowly over time (+0.008 points per month; 95% confidence interval [CI], 0.007‑0.010), whereas after October 2023 declined rapidly (–0.222 points per month; 95% CI, –0.257 to –0.204), indicating a marked reversal in the long-term readability trend (*P* < 0.001). The Flesch Reading Ease differed significantly between the 24-month pre-October and post-October 2023 periods (median, 30.74 [interquartile range, 30.39–31.06] before vs. 28.35 [interquartile range, 27.29–29.54] after; *P* < 0.001). Similar observations were made for other established readability metrics (Flesch–Kincaid Grade Level, Gunning Fog Index, Dale–Chall Readability Score, and Coleman Liau Index) (all *P* < 0.001) (Supplementary Figure 1, available at www.ophthalmologyscience.org).

**Figure 2:**
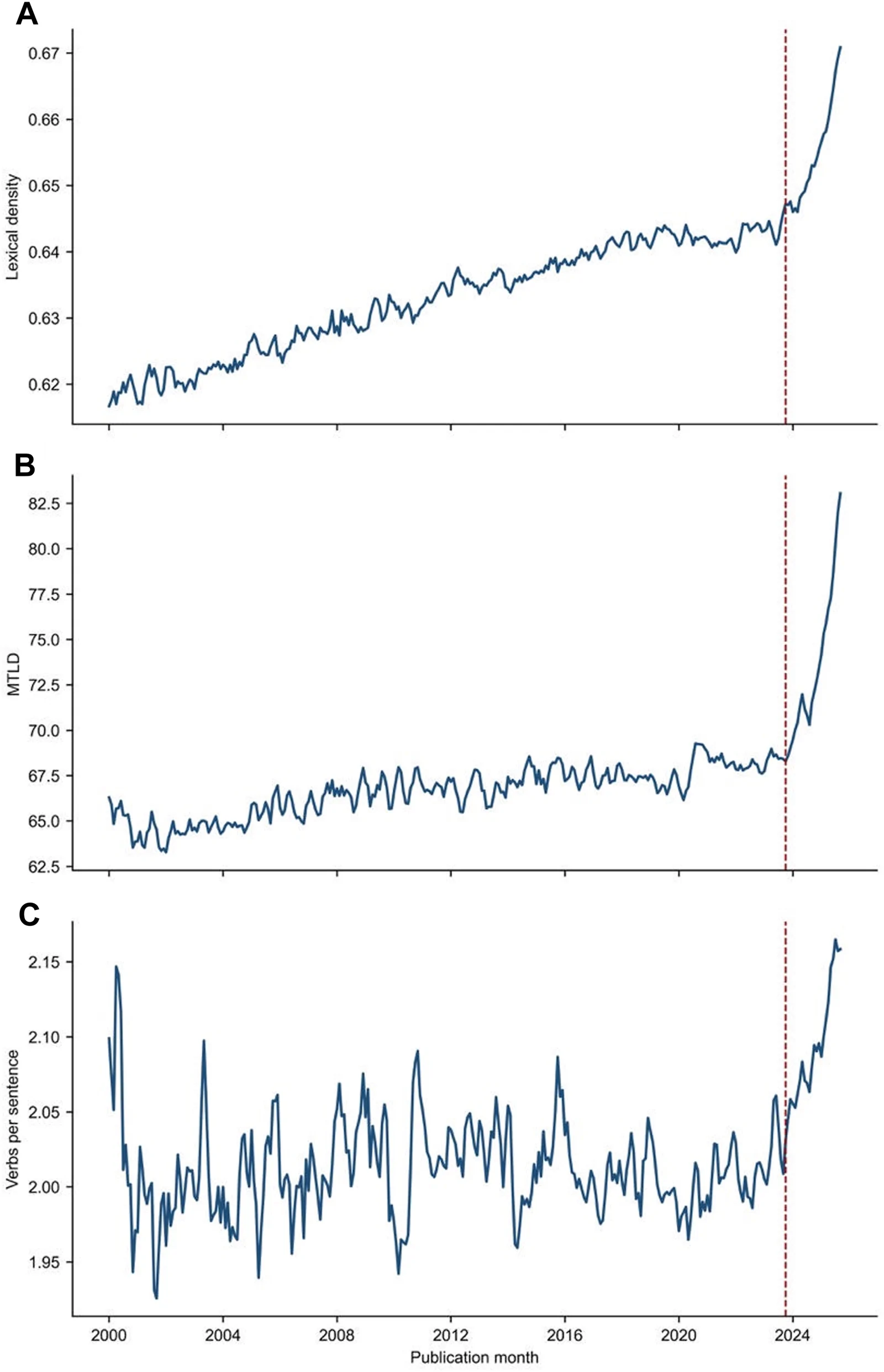
Temporal trends in linguistic features of ophthalmology abstracts. **(A)** Monthly mean lexical density, **(B)** lexical diversity measured by the Measure of Textual Lexical Diversity (MTLD), and **(C)** clause density expressed as verbs per sentence in ophthalmology abstracts. Lines represent centered 3-month rolling means. The vertical red dashed line indicates October 2023.

Temporal changes in linguistic features were assessed using segmented regression with a prespecified breakpoint in October 2023. Lexical density increased slightly before October 2023 (+0.000096/month; 95% CI, 0.000092–0.000101; *P* < 0.001). At the breakpoint, a small decrease was observed (–0.0040; 95% CI, –0.0076 to –0.0004; *P* = 0.029), followed by a significantly steeper increase thereafter (slope change +0.0010; 95% CI, 0.0007–0.0012; *P* < 0.001) (Fig 2A). Lexical diversity (Measure of Textual Lexical Diversity) increased prior to October 2023 (+0.0139/month; 95% CI, 0.012–0.016; *P* < 0.001), with no significant immediate level change at the breakpoint (*P* = 0.055), followed by a significant increase in slope thereafter (slope change +0.557; 95% CI, 0.411–0.703; *P* < 0.001) (Fig 2B). Clause density (verbs per sentence) showed no significant prebreak trend (*P* = 0.89), a small increase at the breakpoint (+0.0208; 95% CI, 0.004–0.038; *P* = 0.015), and a significant increase in slope thereafter (slope change +0.0053; 95% CI, 0.004–0.006; *P* < 0.001) (Fig 2C).

Additional descriptive analyses of underlying linguistic components are shown in Supplementary Figure 2 (available at www.ophthalmologyscience.org). Words per sentence decreased gradually over time without an apparent change at the October 2023 breakpoint, whereas syllables per word and the proportion of polysyllabic words increased sharply after October 2023. Total abstract length increased steadily over time without an abrupt change at the breakpoint. We additionally evaluated monthly hyphen-like character frequency per 1000 words, which did not show a distinct post-2023 change.

**Figure 3:**
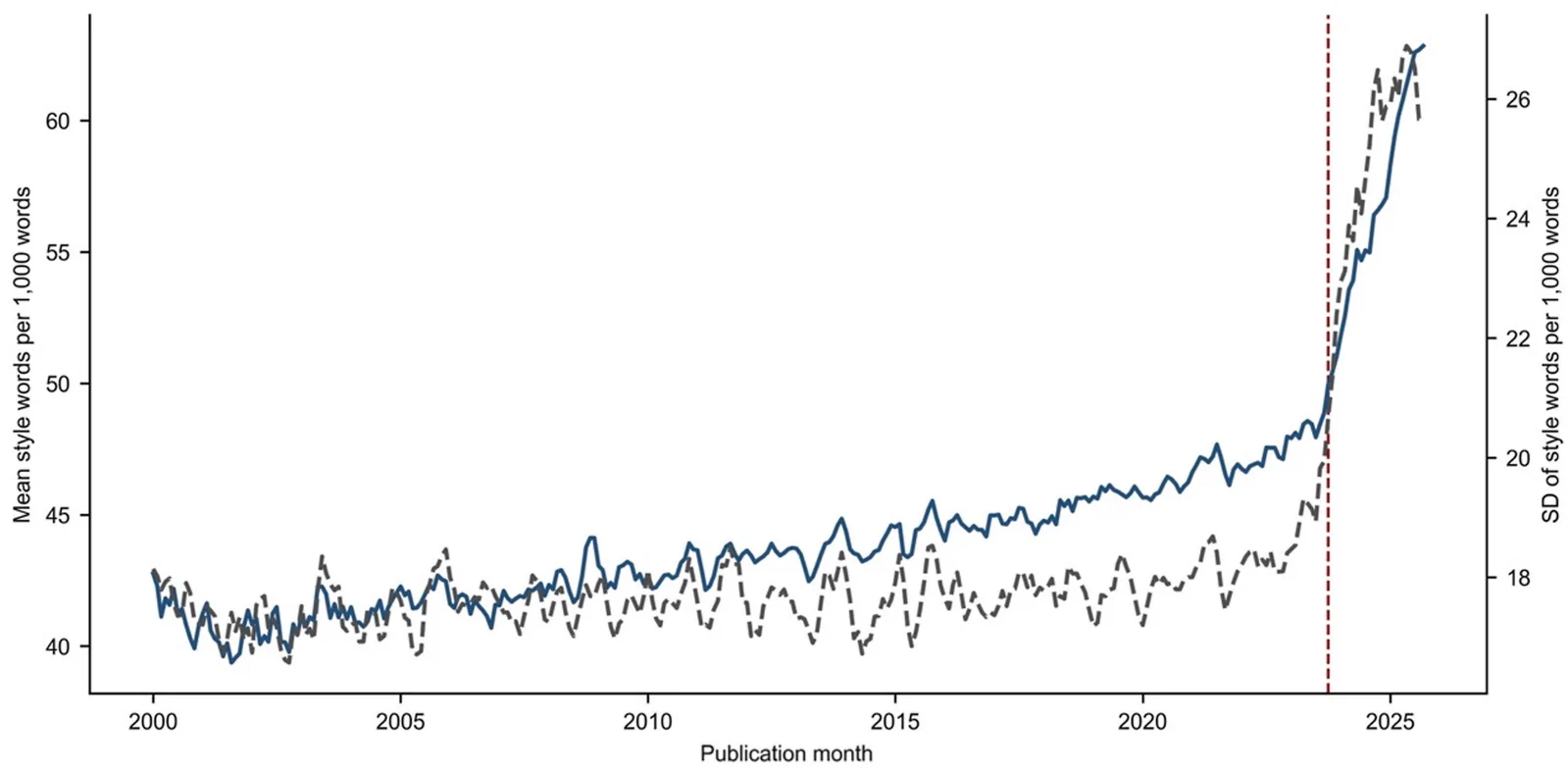
Trend and variability in excessive style-word usage. Monthly mean number of excessive style words per 1000 words (blue solid line, left y-axis) and corresponding standard deviations (gray dashed line, right y-axis) in ophthalmology abstracts. Lines represent centered 3-month rolling means. The vertical red dashed line indicates October 2023.

We adopted the list of “excessive style words” from a recent large-scale analysis of PubMed abstracts between 2010 and 2024 that identified vocabulary showing disproportionate increases in usage following the introduction of LLMs. The relative frequency of these words increased gradually over time, with a marked acceleration after 2023 (Fig 3). At the same time, variability increased substantially. Higher frequencies of these words were associated with lower readability at the monthly level (Pearson r = –0.127, *P* < 0.001).

In a within-author analysis restricted to authors publishing both before and after October 2023 (*n* = 42 333 abstracts; 6418 authors), readability increased prior to the breakpoint (+0.010 points/month; 95% CI, 0.007‑0.012) but declined thereafter (–0.170 points/month), reflecting a significant change in slope (–0.180 points/month; 95% CI, –0.222 to –0.138; *P* < 0.001), with no evidence of a level shift (*P* = 0.56) (Supplementary Figure 3, available at www.ophthalmologyscience.org).

Of the 198 201 publications, 148 993 were classified as structured abstracts. Structured abstracts had higher readability than nonstructured abstracts in the structure-adjusted model (difference, 9.73 points; 95% CI, 9.29–10.18; *P* < 0.001). Before October 2023, readability increased in structured abstracts but decreased in nonstructured abstracts (0.015 vs. –0.015 points/month; interaction, 0.030; 95% CI, 0.027–0.033; *P* < 0.001). After October 2023, readability declined in both groups, with no significant difference in the postbreak slopes between structured and nonstructured abstracts (–0.194 vs. –0.236 points/month; interaction, 0.042; 95% CI, –0.041 to 0.126; *P* = 0.320) (Supplementary Figure 4, available at www.ophthalmologyscience.org).

For analysis of the first-author affiliation country, 167 669 publications with a single first-author country assignment were included. Abstracts from non-native English-speaking countries had higher baseline readability than those from native English-speaking countries (difference, 3.20 points; 95% CI, 2.81–3.59; *P* < 0.001). Before October 2023, readability increased in both groups but more in non-native English-affiliated abstracts (0.008 vs. 0.002 points/month; interaction, 0.0062; 95% CI, 0.004–0.008; *P* < 0.001). After October 2023, readability declined in both groups, with a significantly steeper decline in non-native English-affiliated abstracts (–0.304 vs. –0.095 points/month; interaction, –0.215; 95% CI, –0.280 to –0.149; *P* < 0.001) (Supplementary Figure 5, available at www.ophthalmologyscience.org).

Stratification by journal impact category showed highly similar temporal patterns in higher-impact (Q1–Q2) and lower-impact (Q3–Q4) journals, with no evidence that the post-October 2023 decline in readability differed by journal group (*P* = 0.5) (Supplementary Figure 6, available at www.ophthalmologyscience.org).

## Discussion

The readability of ophthalmology abstracts has undergone a structural shift after more than 2 decades of gradual improvement. Around October 2023, mean readability declined sharply, and readability variability increased, indicating a structural break in what had previously been a steady trajectory. Because readability quantifies the linguistic accessibility of scientific text and shapes how efficiently knowledge is understood and disseminated, a shift has important implications for the communication of research in ophthalmology.

Several studies have reported a decline of readability in scientific abstracts across disciplines over the past decades.^1^^,^^16^^,^^17^ In contrast, we observed the opposite trend within the subfield of ophthalmology, showing an incremental but consistent improvement since 2000. However, the recent reversal of this trend suggests that a recent field-wide change in writing practice may have occurred.

A significant shift in readability was identified around October 2023 using segmented regression with an unknown breakpoint. To better understand the underlying drivers of this change, we examined the linguistic components contributing to readability. Because the Flesch Reading Ease score depends primarily on sentence length and syllables per word, the observed decline can be best interpreted through these components. In our analysis, it was driven predominantly by an increase in syllables per word and in the proportion of polysyllabic words, whereas words per sentence decreased over time (Supplementary Figure 2). Lexical density, lexical diversity, and clause density also increased, indicating a higher proportion of content-bearing words, greater lexical variability, and higher syntactic complexity (Fig 2). The timing of this shift coincides with a broader period of change in scientific writing practices, including the increasing availability of LLM-based language tools, and is consistent with recent reports of linguistic shifts in other academic corpora.^18^ Our findings extend these observations to peer-reviewed ophthalmology journals. However, the present data do not allow causal attribution at the level of individual articles.

Consistent with this pattern, a recent analysis of more than 15 million abstracts demonstrated a disproportionate rise beginning in 2023 in an externally defined vocabulary set linked to recent LLM-era changes in academic writing.^5^ In our corpus of abstracts, the frequency of these excessive style words increased markedly from late 2023 and was associated with worse readability scores.

Additionally, we observed an increase in the variability of readability scores and in the frequency of the externally defined style-word set (Fig 3). This pattern indicates increasing heterogeneity in how abstracts are written and suggests that the observed linguistic shift was not uniform across the literature.

The robustness analyses further argue against a simple compositional explanation. The decline remained evident in a within-author analysis restricted to authors publishing both before and after the breakpoint, indicating that the pattern was not explained solely by turnover in who was publishing. The post-2023 decline also persisted in both structured and nonstructured abstracts, suggesting that differences between abstract formats are unlikely to explain the observed shift. Similar temporal trends were observed across journal impact strata. In country-stratified analyses, the post-2023 decline was present in both abstracts from first-author affiliations in native English-speaking and non-native English-speaking countries, although it was steeper in the latter. Together, these findings suggest that the observed shift was broad-based and not limited to changes in abstract structure, journal mix, authorship, or first-author affiliation country.

Good readability of an abstract ensures the findings are easily understood, which supports the correct evaluation of its innovation and contribution to the field.^1^^,^^18^^,^^19^ This is especially relevant for non-native English speakers, who are disproportionately affected by linguistic complexity.^20^ Considering this, the steeper post-2023 decline observed among abstracts from first-author affiliations in non-native English-speaking countries is notable, although this pattern should be interpreted cautiously because affiliation country is only an imperfect proxy for linguistic background. Recent research has additionally shown that readability is significantly associated with online attention from the general public, which highlights its relevance beyond the academic audience.^21^

These findings may also be relevant to ongoing discussions about artificial intelligence (AI)‑assisted scientific writing. Article-level attribution remains difficult, and available detection approaches have important limitations.^22^^,^^23^ However, Natural Language Processing models could also improve the readability of scientific texts without compromising their meaning.^24^^,^^25^ Therefore, LLMs represent not only an editorial and methodological challenge but also offer a potential opportunity to improve abstract readability and broaden accessibility of scientific knowledge.

This study has limitations. First, because article-level data on AI-assisted writing are not available, the observed readability changes cannot be directly attributed to LLM use. Although many journals require disclosure, these policies rely on self-report and cannot be independently verified, limiting the availability of reliable validation data. In addition, as an observational time-series analysis, the study cannot exclude that other confounding factors may have contributed to the observed changes, such as evolving writing style of authors, changes in editorial practices, and broader shifts in scientific reporting conventions. Second, readability is difficult to quantify and can be perceived slightly differently between individuals. Therefore, readability scores can reflect this only to a certain extent in scientific texts as they analyze linguistic structure.^16^ New approaches with AI-based classifiers understanding the semantic content of texts may generate more accurate readability scores in highly specialized areas like ophthalmology in future research. Nevertheless, the readability metrics used in this study have already been applied and validated in numerous publications over the past decades, thereby supporting their reproducibility and comparability.^14^^,^^15^ Third, syntactic complexity was approximated using verbs per sentence, and automated text processing (textstat and spaCy) may introduce minor inaccuracies. However, as the analysis focuses on relative temporal trends rather than absolute values, these limitations are unlikely to bias the direction of the observed changes. Fourth, country classification relied on first-author affiliation metadata and may not fully reflect an author’s linguistic background. Finally, our extensive corpus of 198 201 abstracts covers a wide selection of ophthalmology journals, but the results may not generalize to other disciplines or non-English publications.

In summary, in this large longitudinal analysis of nearly 200 000 ophthalmology abstracts spanning 25 years, we found a structural reversal in readability beginning in late 2023, accompanied by increased heterogeneity and by a shift toward greater lexical and syntactic complexity. This pattern was evident across multiple readability measures, supported by increases in lexical density, lexical diversity, clause density, syllables per word, and the proportion of polysyllabic words, and was robust to within-author and stratified analyses. In addition, the observed changes were accompanied by an increased use of a predefined set of style-related words. Although these findings occurred during a period of broader change in scientific writing practices, the present study cannot determine their cause. Overall, the results indicate a shift in the linguistic characteristics of ophthalmology abstracts and underline the importance of maintaining readability as a core objective in scientific communication.

## Data Sharing Statement

All data used in this study are publicly available and accessible through the original publication sources. Analytic code was developed for this study and can be shared upon reasonable request.
