# Supplementary material for: A Structural Shift in the Readability of Ophthalmology Abstracts in Late 2023
Source: Ophthalmol Sci. 2026 Jul 28;6(10):101337. doi: 10.1016/j.xops.2026.101337 (PMC13527500; doi:10.1016/j.xops.2026.101337)
Supplement: Supplementary file 1 [file mmc1.pdf]

Supplementary

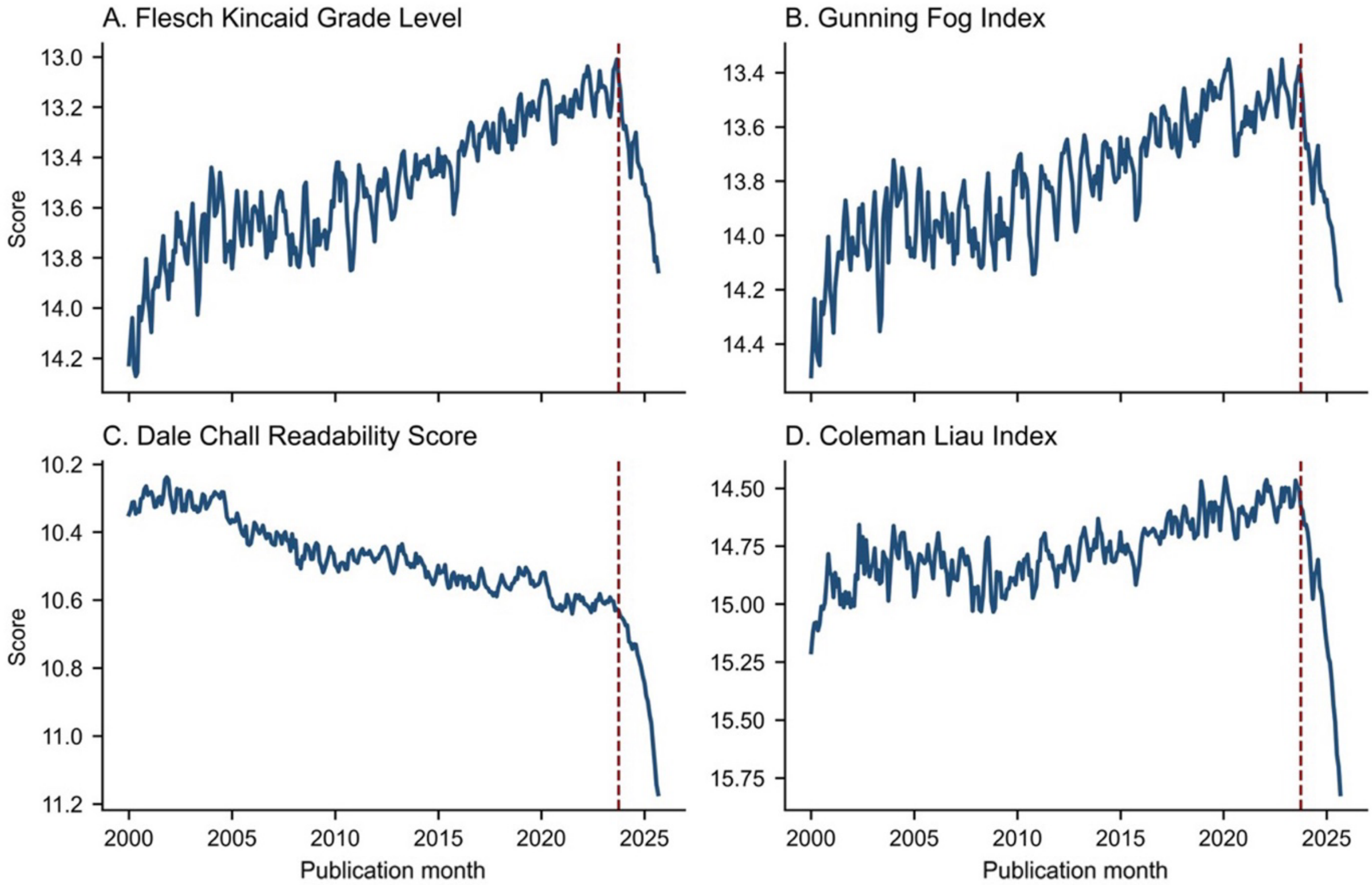

Supplementary Figure 1: Trends in Multiple Readability Indices. Panels show 3-month centered rolling means for (A) Flesch Kincaid Grade Level, (B) Gunning Fog Index, (C) Dale Chall Readability Score, and (D) Coleman Liau Index. The vertical dashed line marks October 2023, corresponding to the breakpoint identified by segmented regression analysis of the Flesch Reading Ease values. To align with Figure 1 (higher = easier), y-axes for Grade Level and Difficulty indices are inverted; downward slopes indicate reduced readability across all panels.

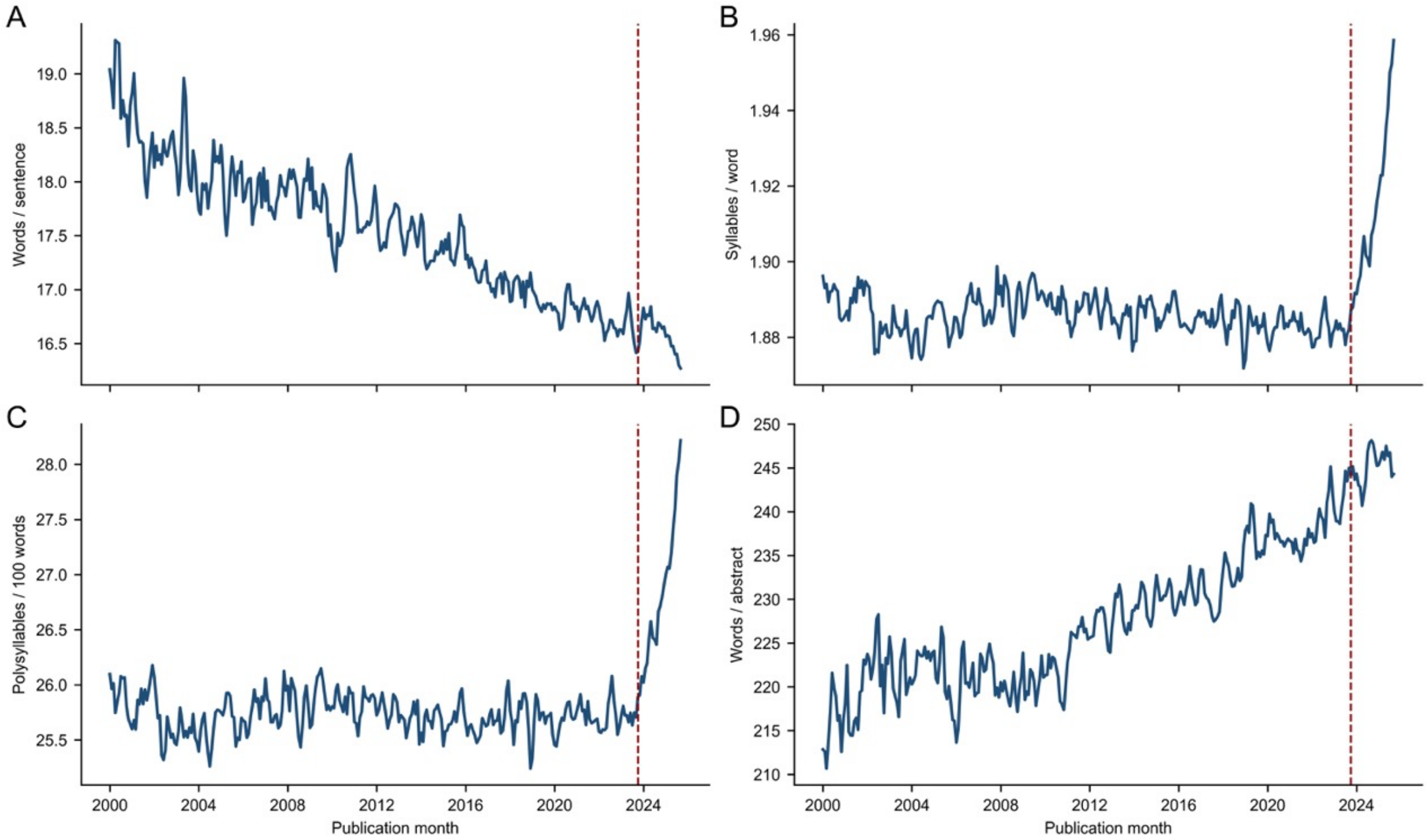

Supplementary Figure 2: Trends in linguistic characteristics of abstracts. Monthly mean (3-month centered rolling average) of (A) words per sentence, (B) syllables per word, (C) polysyllabic words per 100 words, and (D) total words per abstract across the study period. The vertical dashed line indicates October 2023.

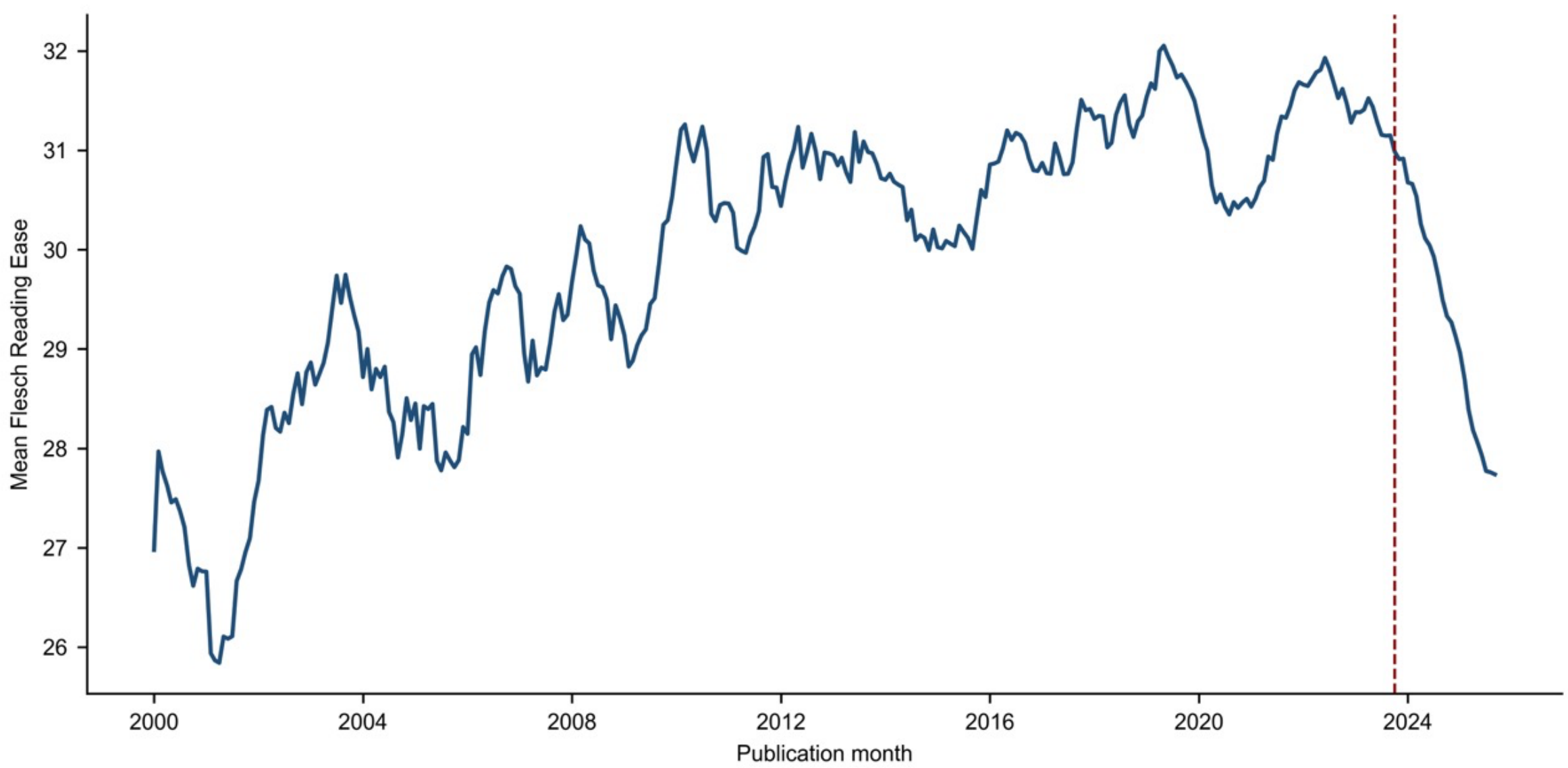

Supplementary Figure 3. Readability trends in the within-author subset. Monthly mean Flesch Reading Ease scores of ophthalmology abstracts in the subset of first authors who published both before and after October 2023 (n=42,333). The line represents a centered 12-month rolling mean. The vertical red dashed line indicates October 2023.

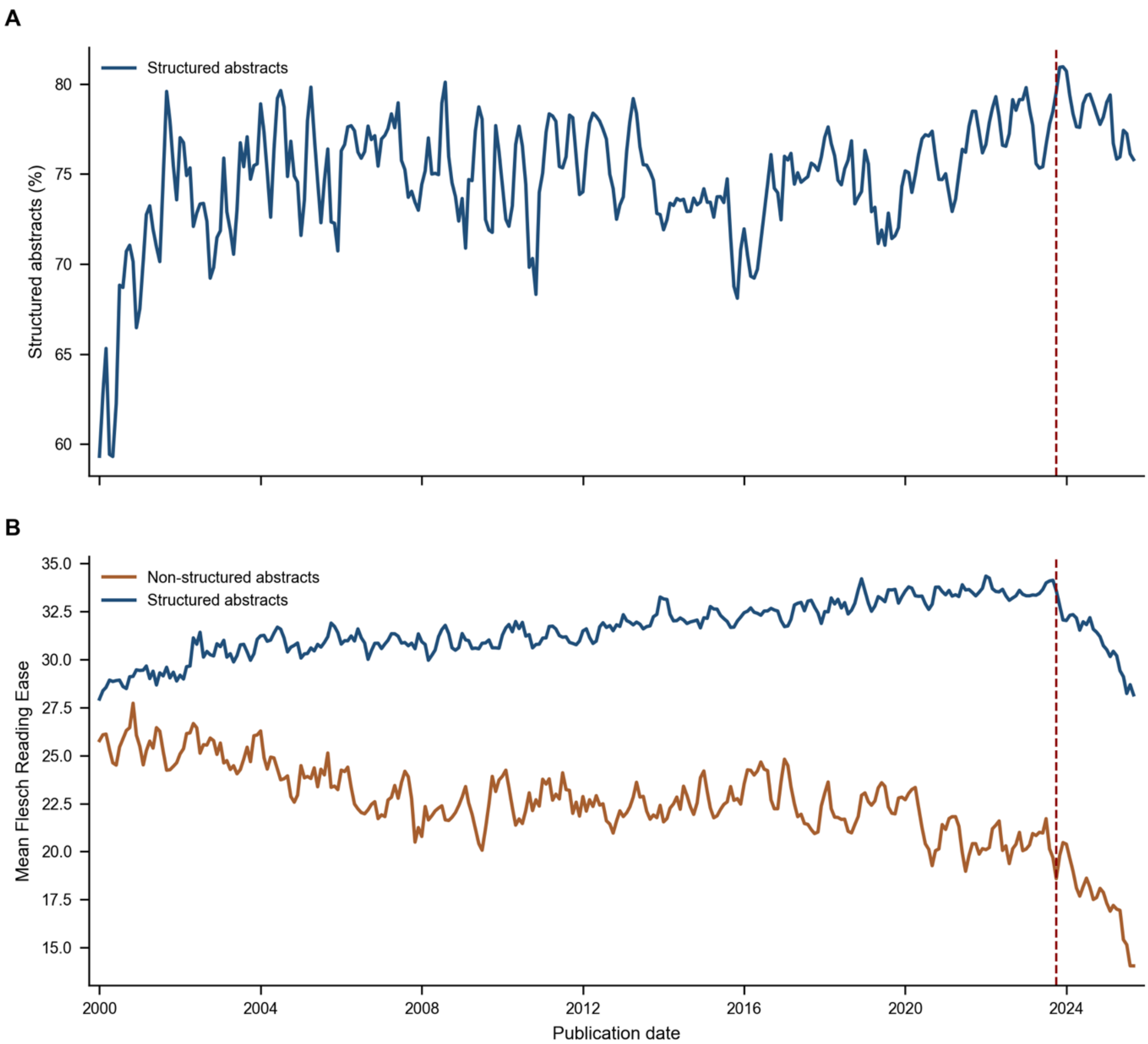

Supplementary Figure 4: Abstract Structure and Readability Over Time. Panels show 3-month centered rolling means for (A) the monthly proportion of structured abstracts and (B) mean Flesch Reading Ease stratified by structured versus non-structured abstracts. The vertical dashed line marks October 2023, corresponding to the breakpoint identified by segmented regression analysis of the Flesch Reading Ease values.

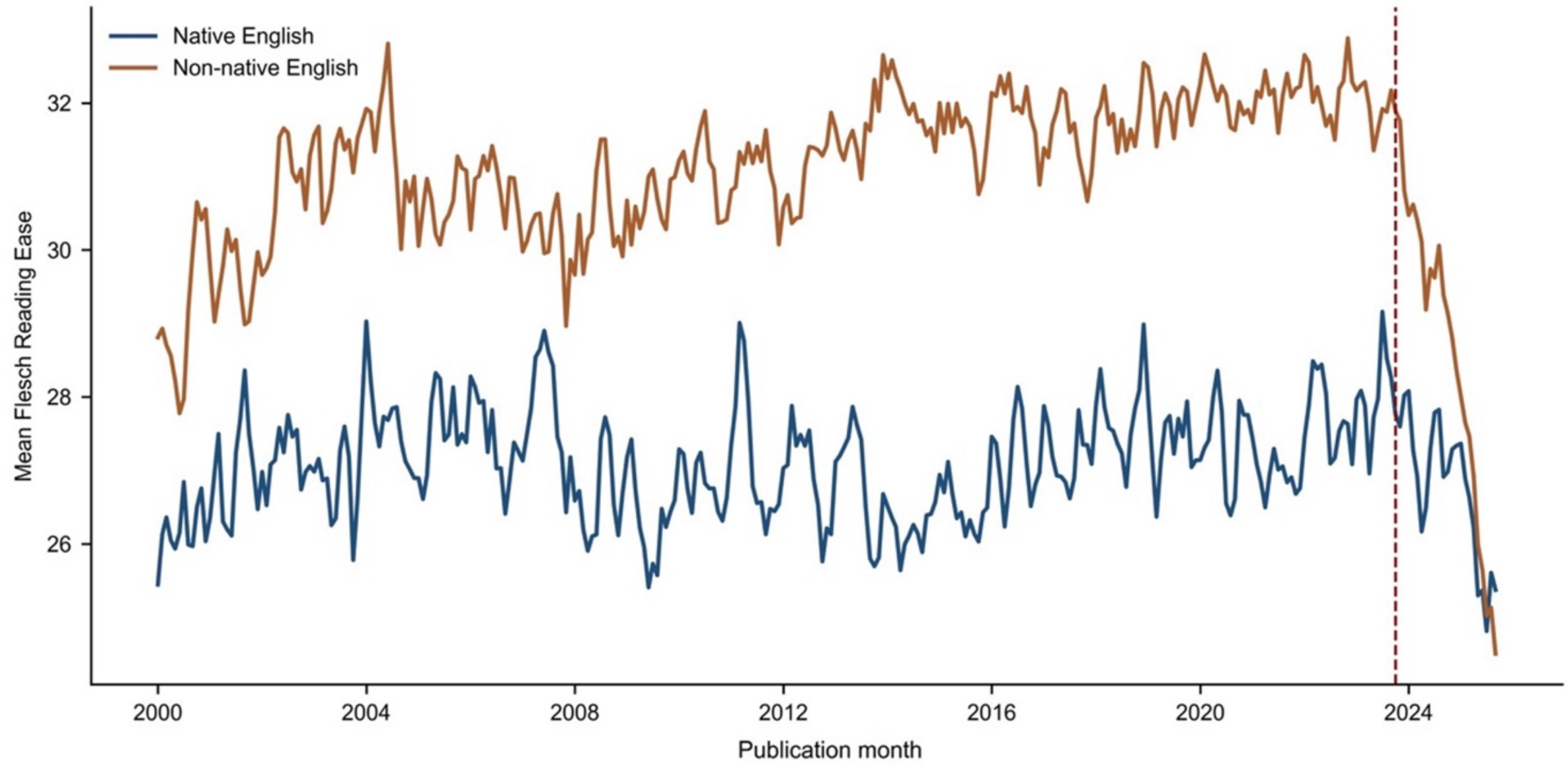

Supplementary Figure 5. Trends in Readability by First-Author Affiliation Language Background. Monthly mean Flesch Reading Ease scores of ophthalmology abstracts stratified by first-author affiliation in native English-speaking versus non–native English-speaking countries. Lines represent centered 3-month rolling means. The vertical red dashed line indicates October 2023.

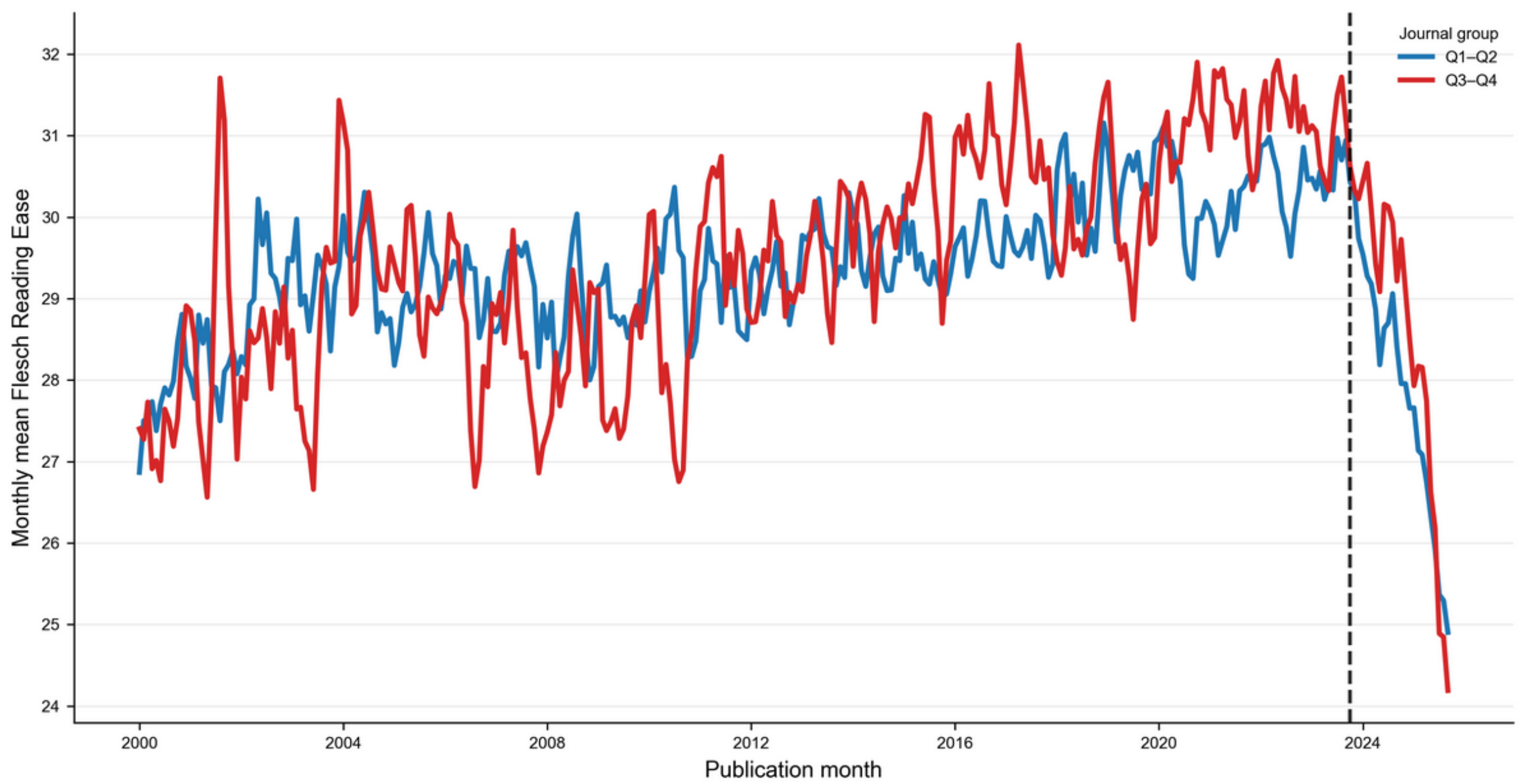

Supplementary Figure 6: Trends in monthly mean Flesch Reading Ease stratified by journal impact group (Q1–Q2 vs Q3–Q4). Lines represent centered 3-month rolling means. The vertical dashed line indicates October 2023.

List of included articles per Journal

Invest Ophthalmol Vis Sci: 17799
Am J Ophthalmol: 8463
Br J Ophthalmol: 7420
Ophthalmology: 6861
J Cataract Refract Surg: 6689
Cornea: 6663
Graefes Arch Clin Exp Ophthalmol: 6658
Exp Eye Res: 6149
Eye (Lond): 5952
Retina: 5655
Clin Ophthalmol: 5597
Eur J Ophthalmol: 5474
Vision Res: 5451
Indian J Ophthalmol: 5064
J Vis: 4882
BMC Ophthalmol: 4743
Int Ophthalmol: 4533
Curr Eye Res: 3974
Acta Ophthalmol: 3760
Mol Vis: 3740
J Glaucoma: 3684
Int J Ophthalmol: 3516
Optom Vis Sci: 3516
Ophthalmic Plast Reconstr Surg: 3336
Ocul Immunol Inflamm: 3076
J Refract Surg: 2903
Transl Vis Sci Technol: 2796
J Ophthalmol: 2793
J AAPOS: 2614
Perception: 2390
Clin Exp Ophthalmol: 2337
Jpn J Ophthalmol: 2315
Ophthalmic Physiol Opt: 2066
J Ocul Pharmacol Ther: 1962
Can J Ophthalmol: 1959
Eye Contact Lens: 1841
Curr Opin Ophthalmol: 1823
Clin Exp Optom: 1782
JAMA Ophthalmol: 1742
Ophthalmic Res: 1631
Ophthalmologica: 1591
Cont Lens Anterior Eye: 1502
Semin Ophthalmol: 1499
Ophthalmic Epidemiol: 1418
Surv Ophthalmol: 1333
J Neuroophthalmol: 1278

Doc Ophthalmol: 1276
Ophthalmol Ther: 1134
Ophthalmol Retina: 1090
Ocul Surf: 991
Cutan Ocul Toxicol: 973
J Ophthalmic Vis Res: 944
Prog Retin Eye Res: 909
Asia Pac J Ophthalmol (Phila): 830
Ophthalmol Sci: 720
BMJ Open Ophthalmol: 720
Int J Retina Vitreous: 657
J Curr Ophthalmol: 591
J Ophthalmic Inflamm Infect: 575
Ophthalmol Glaucoma: 490
Eye Vis (Lond): 433
J Optom: 407
J Eye Mov Res: 331
Ther Adv Ophthalmol: 297
Annu Rev Vis Sci: 233
Clin Optom (Auckl): 224
Eye Brain: 146

Supplementary Table S1: List of Excess Vocabulary Words: The list of excess style words is reproduced verbatim from Kobak & González-Márquez (2024) [arXiv:2406.07016] and the associated GitHub repository (MIT License). © 2024 Dmitry Kobak and Rita González-Márquez.

**Word**	**Type**	**Part_of_Speech**
accentuates	style	verb
achieving	style	verb
acknowledges	style	verb
acknowledging	style	verb
across	style	preposition
additionally	style	adverb
address	style	verb
addresses	style	verb
addressing	style	verb
adept	style	adjective
adhered	style	verb
adhering	style	verb
advancement	style	noun
advancements	style	noun
advancing	style	verb
advocates	style	verb
advocating	style	verb

affirming	style	verb
afflicted	style	verb
aiding	style	verb
aims	style	verb
akin	style	adjective
align	style	verb
aligning	style	verb
aligns	style	verb
alongside	style	preposition
amid	style	preposition
amidst	style	preposition
analysis	style	noun
announced	style	verb
apologizes	style	verb
approach	style	noun
assess	style	verb
assessed	style	verb
assessing	style	verb
assessments	style	noun
attains	style	verb
attributed	style	verb
augmenting	style	verb
avenue	style	noun
avenues	style	noun
based	style	verb
between	style	preposition
bolster	style	verb
bolstered	style	verb
bolstering	style	verb
both	style	pronoun/adverb
broader	style	adjective
burgeoning	style	verb
capabilities	style	noun
capitalizing	style	verb
categorized	style	verb
categorizes	style	verb
categorizing	style	verb
challenge	style	verb
challenges	style	noun
combating	style	verb
commendable	style	adjective
compelling	style	adjective
complex	style	noun

complicates	style	verb
complicating	style	verb
comprehend	style	verb
comprehending	style	verb
comprehensive	style	adjective
comprising	style	verb
conditions	style	noun
conducted	style	verb
consequently	style	verb
consolidates	style	verb
contributing	style	verb
conversely	style	adverb
correlating	style	verb
crafted	style	verb
crafting	style	verb
crucial	style	adjective
culminating	style	verb
customizing	style	verb
declare	style	verb
declared	style	verb
deductively	style	adverb
delineates	style	verb
delve	style	verb
delved	style	verb
delves	style	verb
delving	style	verb
demonstrated	style	verb
demonstrates	style	verb
demonstrating	style	verb
dependability	style	noun
dependable	style	adjective
despite	style	preposition
detailing	style	verb
detrimentally	style	adverb
diminishes	style	verb
diminishing	style	verb
discern	style	verb
discerned	style	verb
discernible	style	adjective
discerning	style	verb
displaying	style	verb
disrupts	style	verb
distinct	style	adjective

distinctions	style	noun
distinctive	style	adjective
diverse	style	adjective
during	style	preposition
easing	style	verb
effectively	style	adverb
elevate	style	verb
elevated	style	verb
elevates	style	verb
elevating	style	verb
elucidate	style	verb
elucidates	style	verb
elucidating	style	verb
embracing	style	verb
emerged	style	verb
emerges	style	verb
emphasises	style	verb
emphasising	style	verb
emphasize	style	verb
emphasizes	style	verb
emphasizing	style	verb
employed	style	verb
employing	style	verb
employs	style	verb
empowers	style	verb
emulating	style	verb
emulation	style	noun
enabling	style	verb
encapsulates	style	verb
encompass	style	verb
encompassed	style	verb
encompasses	style	verb
encompassing	style	verb
endangering	style	verb
endeavors	style	noun
endeavours	style	noun
enduring	style	verb
enhance	style	verb
enhanced	style	verb
enhancements	style	noun
enhances	style	verb
enhancing	style	verb
ensuring	style	verb

equipping	style	verb
escalating	style	verb
essentials	style	noun
evaluates	style	verb
evolving	style	verb
exacerbating	style	verb
examines	style	verb
exceeding	style	verb
excels	style	verb
exceptional	style	adjective
exceptionally	style	adverb
exerting	style	verb
exhibit	style	verb
exhibited	style	verb
exhibiting	style	verb
exhibits	style	verb
expedite	style	verb
expediting	style	verb
exploration	style	noun
explores	style	verb
facilitated	style	verb
facilitates	style	verb
facilitating	style	verb
featuring	style	verb
fight	style	noun/verb
findings	style	noun
focusing	style	verb
formidable	style	adjective
fostering	style	verb
fosters	style	verb
foundational	style	adjective
furnish	style	verb
garnered	style	verb
garnering	style	verb
gauged	style	verb
grappling	style	verb
groundbreaking	style	adjective
groundwork	style	noun
hardest	style	noun
harness	style	verb
harnesses	style	verb
harnessing	style	verb
heighten	style	verb

heightened	style	adjective
highlight	style	verb
highlighting	style	verb
highlights	style	verb
hinder	style	verb
hinges	style	verb
hinting	style	verb
hold	style	verb
holds	style	verb
however	style	adverb
identified	style	verb
illuminates	style	verb
illuminating	style	adjective
imbalances	style	verb
impact	style	noun
impactful	style	adjective
impacting	style	verb
impede	style	verb
impeding	style	adjective
imperative	style	adjective
impressive	style	adjective
inadequately	style	adverb
including	style	preposition
incorporates	style	verb
incorporating	style	verb
indicating	style	verb
individuals	style	noun
influencing	style	verb
inherent	style	adjective
initially	style	adverb
innovative	style	adjective
inquiries	style	noun
insights	style	noun
integrates	style	verb
integrating	style	verb
integration	style	noun
interconnectedness	style	noun
interplay	style	noun
into	style	preposition
intricacies	style	noun
intricate	style	adjective
intricately	style	adverb
introduces	style	adjective

invaluable	style	adjective
investigates	style	verb
involves	style	verb
involving	style	verb
juxtaposed	style	adjective
leading	style	verb
leverages	style	verb
leveraging	style	verb
like	style	particle
limitations	style	noun
linked	style	verb
maintaining	style	verb
merges	style	verb
methodologies	style	noun
meticulous	style	adjective
meticulously	style	adverb
midst	style	preposition
multifaceted	style	adjective
necessitate	style	verb
necessitates	style	verb
necessitating	style	verb
necessity	style	noun
need	style	verb
notable	style	adjective
notably	style	advebr
noteworthy	style	adjective
nuanced	style	adjective
nuances	style	noun
observed	style	verb
offer	style	verb
offering	style	verb
offers	style	verb
optimizing	style	verb
orchestrating	style	verb
outcomes	style	noun
outlines	style	verb
overlook	style	verb
overlooking	style	verb
overwhelmed	style	verb
particularly	style	adverb
paving	style	verb
persist	style	verb
pinpoint	style	verb

pinpointed	style	verb
pinpointing	style	verb
pioneering	style	verb
pioneers	style	noun
pivotal	style	adjective
poised	style	verb
pose	style	verb
posed	style	verb
poses	style	verb
posing	style	verb
postponed	style	verb
potential	style	adjective
potentially	style	adverb
precise	style	adjective
predominantly	style	adverb
presents	style	verb
preserving	style	verb
pressing	style	adjective
prevalent	style	adjective
primarily	style	adverb
primary	style	adjective
promise	style	verb
promising	style	verb
pronounced	style	adjective
propelling	style	verb
providing	style	verb
realizes	style	verb
realm	style	noun
realms	style	noun
recognizing	style	verb
refine	style	verb
refines	style	verb
refining	style	verb
reframing	style	verb
remains	style	verb
remarkable	style	adjective
renowned	style	adjective
research	style	noun
resulting	style	verb
rethink	style	verb
revealed	style	verb
revealing	style	verb
reveals	style	verb

revolutionize	style	verb
revolutionizing	style	verb
revolves	style	verb
role	style	noun
scrutinize	style	verb
scrutinized	style	verb
scrutinizing	style	verb
seamless	style	adjective
seamlessly	style	adverb
seeks	style	verb
serves	style	verb
serving	style	verb
shaping	style	verb
shedding	style	verb
showcased	style	verb
showcases	style	verb
showcasing	style	verb
signifying	style	verb
solidify	style	verb
spanned	style	verb
spanning	style	verb
specifically	style	adverb
spurred	style	verb
stands	style	verb
statement	style	noun
stemming	style	verb
strategically	style	adjective
strategies	style	noun
streamline	style	verb
streamlined	style	verb
streamlines	style	verb
streamlining	style	verb
struggle	style	verb
subsequently	style	adverb
substantial	style	adjective
substantiated	style	verb
substantiates	style	verb
surged	style	verb
surmount	style	verb
surpass	style	verb
surpassed	style	verb
surpasses	style	verb
surpassing	style	verb

swift	style	adjective
swiftly	style	adverb
techniques	style	noun
their	style	pronoun
thereby	style	adverb
these	style	pronoun
this	style	pronoun
thorough	style	adjective
through	style	preposition
transformative	style	adjective
typically	style	adverb
ultimately	style	adverb
uncharted	style	adjective
uncovering	style	verb
underexplored	style	verb
underscore	style	verb
underscored	style	verb
underscores	style	verb
underscoring	style	verb
understanding	style	verb
unexplored	style	adjective
unlocking	style	verb
unparalleled	style	adjective
unraveling	style	verb
unveil	style	verb
unveiled	style	verb
unveiling	style	verb
unveils	style	verb
uphold	style	verb
upholding	style	verb
urging	style	verb
using	style	verb
utilized	style	verb
utilizes	style	verb
utilizing	style	verb
valuable	style	adjective
various	style	adjective
varying	style	verb
verifies	style	verb
versatility	style	noun
wandering	style	verb
warranting	style	verb
were	style	verb

while	style	preposition
within	style	adverb
yielding	style	verb
